# *Listeria monocytogenes* in ready to eat meat products from Zambia: phenotypical and genomic characterization of isolates

**DOI:** 10.3389/fmicb.2023.1228726

**Published:** 2023-08-30

**Authors:** Gabriella Centorotola, Maureen Wakwamba Ziba, Alessandra Cornacchia, Alexandra Chiaverini, Marina Torresi, Fabrizia Guidi, Cesare Cammà, Benson Bowa, Samson Mtonga, Phelly Magambwa, Nicola D’Alterio, Massimo Scacchia, Francesco Pomilio, Geoffrey Muuka

**Affiliations:** ^1^Istituto Zooprofilattico Sperimentale dell’Abruzzo e del Molise “G. Caporale”, Teramo, Italy; ^2^Central Veterinary Research Institute, Ministry of Fisheries and Livestock, Lusaka, Zambia

**Keywords:** ready to eat, meat products, African countries, *Listeria monocytogenes*, whole genome sequencing

## Abstract

The contamination of ready to eat foods (RTE) products due to *Listeria monocytogenes* could compromise the products safety becoming a great risk for the consumers. The high presence of *L. monocytogenes* in RTE products has been described worldwide, but few data are available about these products from African countries. The aims of this study were to report the presence of *L. monocytogenes* in Zambian RTE products, providing genomic characterization and data on similarity with African circulating strains using whole genome sequencing (WGS). A total of 304 RTE products, produced by different Zambian manufacturers, were purchased at retail, from major supermarkets located in Lusaka, Zambia, comprising 130 dairy and 174 meat products. *L. monocytogenes* was detected only in 18 (10.3%) RTE meat products of the 174 samples tested. The MLST analysis grouped the 18 *L. monocytogenes* isolates in 7 clonal complexes (CCs): CC1 (*n* = 5), CC2 (*n* = 4), CC9 (*n* = 4), CC5 (*n* = 2), CC121 (*n* = 1), CC155 (*n* = 1), and CC3 (*n* = 1). According to the cgMLST results, several clusters were detected, in particular belonging to hyper-virulent clones CC1 and CC2. Regarding the virulence factors, a complete *L. monocytogenes* Pathogenicity Island 3 (LIPI-3) was present both in the CC1 and CC3, in addition to LIPI-1. Several resistance genes and mobile genetic elements were detected, including Stress Islands, the *bcrABC* cassette and *Tn6188*_qac transposon, plasmids and intact prophages. Despite being a first preliminary work with a limited number of samples and isolates, this study helped to increase existing knowledge on contaminated RTE products in Zambia, confirming the presence of hyper-virulent *L. monocytogenes* CCs, which could play an important role in human diseases, posing a public health concern for consumers.

## 1. Introduction

The consumption of convenience foods, such as sliced and prepackaged ready to eat (RTE) products, has increased worldwide. The RTE meat and dairy products require no post-processing heating or other additional antimicrobial treatments before consumption, hence the contamination of pathogenic microorganisms, such as *Listeria monocytogenes,* could compromise the safety of the products becoming a great risk for the final consumers (Calvo-Arrieta et al., 2021).

The presence of *L. monocytogenes* in RTE products has been worldwide described and confirmed, including European Union (European Food Safety Authority, 2022) and South Africa (SA) (Matle et al., 2019). The contamination of RTE products can occur along the entire food production chain as *L. monocytogenes* is widely distributed in primary production areas and in food production environments (FPE) of food producing plants (FPP), processing and packaging plants, storage facilities and retail shops (Gupta and Achyut, 2022). *L. monocytogenes* is ubiquitous and able to survive at low refrigeration temperatures, low pH and high salt concentrations, but also to develop biofilms, increasing the risks of food contamination (Lee et al., 2019; Osek and Wieczorek, 2022). *L. monocytogenes* contamination is associated with poor food processing practices and cross-contaminations in FPP, involving mainly RTE, such as vegetables, fish, dairy and meat products (Dufailu et al., 2021).

*Listeria monocytogenes* is a foodborne pathogen that can cause listeriosis, a potentially severe foodborne disease in human and animals (Maury et al., 2019). In healthy people, more frequently *L. monocytogenes* is a cause a febrile gastroenteritis, whereas in susceptible people, such as children, elderly, immunocompromised and pregnant women, it may lead to septicemia, meningitis, abortion and eventually death (Osek and Wieczorek, 2022). Even if rare, an occurrence of respiratory infection caused by *L. monocytogens* has been reported in Italy (Guidi et al., 2021a).

The European Food Safety Authority (European Food Safety Authority, 2022) reported 2,183 confirmed invasive human cases of *L. monocytogenes* in 2021. Moreover, the European case fatality rate is high (13.7%), similar to 2020 (European Food Safety Authority, 2022), confirming listeriosis as one of the most severe foodborne diseases. *L. monocytogenes* outbreaks reported were linked to different food matrices, such as ice cream (Chen et al., 2017b), cheese (Chen et al., 2017a), RTE fish products (Schjørring et al., 2017) and RTE meat products (Althaus et al., 2017; Duranti et al., 2018; Gelbíčová et al., 2018; Orsini et al., 2018).

Few data are available regarding the prevalence of *L. monocytogenes* in RTE food products from African countries (Garedew et al., 2015; Oyinloye, 2016; Matle et al., 2019), however, one of the biggest listeriosis outbreak, with 1,060 cases, was reported in SA between 2017 and 2018, due to polony, a RTE processed meat product (Smith et al., 2019; Thomas et al., 2020).

The aims of this study were to report the presence of *L. monocytogenes* in Zambian RTE products, provide data on the genomic characterization of isolated strains and evaluate their similarity with strains circulating in other African countries using whole genome sequencing (WGS).

## 2. Materials and methods

### 2.1. Sample collection and *Listeria monocytogenes* detection

Between October 2019 and February 2020, a total of 304 RTE Zambian products were collected; all the products were randomly selected and purchased at retail, from the major supermarkets located in Lusaka, Zambia. The RTE products considered in this study are usually consumed in the cities which has more wealthy people who prefer easy and fast made foods, hence the choice of selection site. The RTE product categories included 130 dairy products (milk drink *n* = 94, cheese *n* = 18, ice cream *n* = 16, butter *n* = 2) and 174 meat products (polony *n* = 84, ham *n* = 43, sausage *n* = 21, biltong *n* = 15, and salami *n* = 11). All products were produced in Zambia by local manufacturers, in particular: 16 different meat (M) and 23 different dairy (D) manufacturers. The sample identification, product type, RTE category, data of sampling and Zambian producer manufacturer were reported in Supplementary Table S1.

From the supermarkets, the packaged samples were placed in a cool box with ice, kept at 8°C and transported to the Central Veterinary Research Institute (CVRI) laboratory on the same day. The RTE products were processed immediately on arrival at the laboratory and tested for *L. monocytogenes* detection, according to ISO (International Organization for Standardization) (2017). Briefly, 25 g of each sample was cultured in 225 mL of Half Fraser enrichment broth (Oxoid, Hampshire, England) for 24 h ± 2 h at 30°C ± 1°C. After the incubation, 0.1 mL of the culture was poured into 10 mL of Fraser broth (Oxoid, Hampshire, England), incubated for 24 h ± 2 h at 37°C ± 1°C. A loopful of both culture suspensions were streaked on to two selective media plates: Agar Listeria according to Ottaviani and Agosti (ALOA) agar (Biolife, Milan, Italy) and Oxford agar (Oxoid, Hampshire, England) and both incubated at 37°C ± 1°C for 24–48 h. Suspect presumptive colonies of *L. monocytogenes* (small, convex bluish-green colonies with opaque white halo on ALOA plate and gray colonies with black zones and sunken center on Oxford plate) were selected in order to perform all confirmation tests reported in the ISO11290-1:2017 (i.e., hemolysis, sugar fermentation). All the *L. monocytogenes* isolates were stored in microbank tubes (Pro Lab Diagnostics, TX, USA) at −20°C and sent, with frozen transport, to the Italian National Reference Laboratory for *L. monocytogenes* (NRL-*Lm*) of Istituto Zooprofilattico Sperimentale dell’Abruzzo e del Molise (IZSAM) for further phenotypical and genomic characterization.

### 2.2. Antimicrobial susceptibility testing

All the *L. monocytogenes* isolates were tested for antimicrobial susceptibility (AST) by broth microdilution method with the Sensititre OptiRead Automated Fluorometric Plate Reading System (Thermo Scientific, Monza, Italy), in order to verify the susceptibility profile of *L. monocytogenes* strains. The minimum inhibitory concentration (MIC) was *in vitro* assessed using the *Haemophilus* spp. and *Streptococcus pneumoniae* Sensititre plate HPB1 (Thermo Scientific, Monza, Italy).

*Listeria monocytogenes* isolates, stored in microbank tubes (Pro Lab Diagnostics, TX, USA) in the NRL-*Lm* at −80°C, were cultured in Brain Heart Infusion (BHI) broth for 20–24 h at 37°C ± 1°C. A loop was streaked on blood agar plates (Liofilchem, Roseto degli Abruzzi, Teramo, Italy) and incubated at 37°C ± 1°C for 24 h ± 1 h. At the end of the incubation, the suspensions and Sensititre plates HPB1 were prepared according to the Sensititre plate guide booklet.^1^ The Sensititre plates HPB1 were incubated at 37°C ± 1°C for 20–24 h, and immediately after, the MIC values were manually read using the Sensititre Vizion Digital MIC Viewing System (Thermo Scientific, Monza, Italy). The interpretation of MIC results was carried out in accordance with EUCAST guidelines [EUCAST (European Committee on Antimicrobial Susceptibility Testing), 2021]. If no specific EUCAST breakpoints were available, results were interpreted considering as reference *Enterococcus* spp. and *Streptococcus pneumoniae,* according to Fischer et al. (2020) (Supplementary Table S2). The MIC value of cefixime was inferred based on cefuroxime susceptibility considering as reference *Streptococcus pneumoniae*. All the extended dilution range of tested antimicrobials are summarized in Supplementary Table S3.

### 2.3. Whole genome sequencing and bioinformatics analysis

*Listeria monocytogenes* isolates, stored in microbank tubes (Pro Lab Diagnostics, Round Rock, TX, USA) in the NRL-*Lm* at −80°C, were firstly streaked on ALOA agar (Liofilchem, Roseto degli Abruzzi, Teramo, Italy), incubated at 37°C ± 1°C for 24–48 h. The DNA extraction was performed on each strain according to Portmann et al. (2018), with minor modifications, firstly adding lysozyme from chicken egg solution (20 mg/mL) (Sigma-Aldrich, Milan, Italy) and, then, using the QIAamp DNA Mini Kit (Qiagen Hilden, Germany), following the manufacturer’s protocol. The purity of the extracts was evaluated by Biospectrometer fluorescence (Eppendorf, Milan, Italy), measuring the absorbance (A), in particular A260/280 and A260/230 values.

Starting from 1 ng of input DNA, the Nextera XT DNA chemistry (Illumina, San Diego, CA) was used for library preparation, according to the manufacturer’s protocols. WGS was performed on the NextSeq 500 platform (Illumina, San Diego, CA, US) with the NextSeq 500/550 mid output reagent cartridge v2 (300 cycles, standard 150-bp paired-end reads).

For the WGS data analysis, an in-house pipeline (Cito et al., 2018) was used which included steps for trimming (Trimmomatic v0.36) (Bolger et al., 2014) and quality control check of the reads (FastQC v0.11.5) (Wingett and Andrews, 2018). Genome *de novo* assembly of paired-end reads was performed using SPAdes v3.11.1 (Bankevich et al., 2012) with default parameters for the Illumina platform 2 × 150 chemistry (−only-assembler –careful –k21, 33, 55, 77). Subsequently, the genome assembly quality check was performed with QUAST v.4.3 (Gurevich et al., 2013). The genomes quality was checked according to the parameters recommended by Timme et al. (2020).

The multilocus sequence typing (MLST) and core genome multilocus sequence typing (cgMLST) analysis were performed according to Pasteur’s reference schemes.^2^ The threshold of ≤7 allelic distance (AD) was considered for cgMLST cluster definition (Moura et al., 2016). The software GrapeTree (Zhou et al., 2018) was used to obtain the Minimum Spanning tree (MSTreeV2).

The *L. monocytogenes* genomes in this study were also characterized *in silico* using BIGSdb-*Lm* database tools (accessed on February 2022), querying for: “*Virulence*,” “*Antibiotic resistance*,” “*Metal and disinfectant resistance*” and “*Stress Islands*.” The identification of mobile genetic elements, such as prophages and plasmids, was performed using PHASTER (accessed on February 2022)^3^ and PlasmidFinder 2.1 (accessed on February 2022)^4^ platforms, respectively.

In addition, a genomic comparison based on AD was performed between *L. monocytogenes* genomes and genomic data available at the National Center for Biotechnology Information (NCBI), related to previous studies on food, environmental and clinical samples from African countries.

The genome assemblies were deposited at DDBJ/ENA/GenBank under the BioProject PRJNA965923.

## 3. Results

### 3.1. *Listeria monocytogenes* detection

Out of the 304 RTE food tested, *L. monocytogenes* was detected in 18 (10.3%) of the 174 RTE meat products samples and none from dairy products. Data in detail were reported in the Table 1.

**Table 1:** Identification of 18 samples positive for *L. monocytogenes* and related ready to eat category, local producer manufacturer, BioSample accession number and clonal complex results of the related *L. monocytogenes* isolates.

Sample identification	Strain identification	BioSample accession	Ready to eat category	Local manufacturer	Clonal complex
27	2021.TE.2	SAMN34509205	ham	M1	CC1
28	2021.TE.3	SAMN34509206	ham	M1	CC1
31	2021.TE.4	SAMN34509207	salami	M1	CC1
36	2021.TE.5	SAMN34509208	polony	M2	CC2
39	2021.TE.6	SAMN34509209	sausage	M2	CC2
66	2021.TE.7	SAMN34509210	polony	M3	CC155
80	2021.TE.8	SAMN34509211	sausage	M4	CC9
84	2021.TE.9	SAMN34509212	polony	M2	CC2
106	2021.TE.10	SAMN34509213	polony	M5	CC2
108	2021.TE.11	SAMN34509214	ham	M1	CC1
115	2021.TE.12	SAMN34509215	polony	M6	CC5
118	2021.TE.13	SAMN34509216	polony	M1	CC5
135	2021.TE.14	SAMN34509217	biltong	M7	CC1
150	2021.TE.15	SAMN34509218	sausage	M8	CC9
154	2021.TE.16	SAMN34509219	polony	M8	CC9
162	2021.TE.17	SAMN34509220	biltong	M7	CC3
164	2021.TE.18	SAMN34509221	ham	M9	CC121
169	2021.TE.19	SAMN34509222	polony	M8	CC9

The presence of *L. monocytogenes* in RTE meat products was observed in polony samples (*n* = 8), followed by ham (*n* = 4), sausage (*n* = 3), biltong (*n* = 2), and salami (*n* = 1). Among all the brands considered, more than one positive sample were detected in products of M1 (*n* = 5), M8 (*n* = 3), M2 (*n* = 3), and M7 (*n* = 2). For each positive sample, one *L. monocytogenes* colony was picked for the characterization, for a total of 18 isolates.

### 3.2. Antimicrobial susceptibility testing

**Figure 1:**
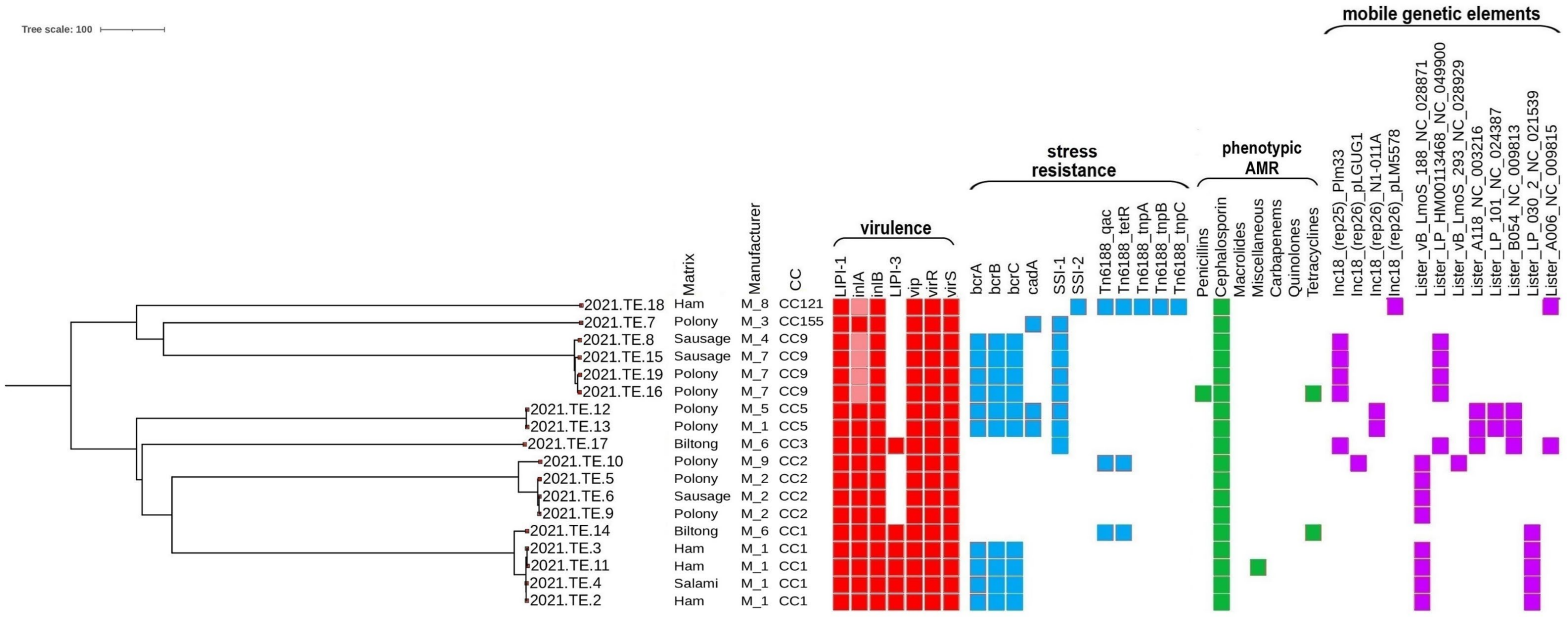
Core genome multilocus sequence typing (cgMLST) phylogenetic tree grouping of virulence, resistance, phenotypic antimicrobial resistance and mobile genetic elements across the 18 *L. monocytogenes* isolates. Virulence (red square), resistance (blue square), phenotypic antimicrobial resistance (green square), and mobile genetic elements (purple square) are shown in the heatmap. Colored square: presence of the gene or phenotypic resistance; light red: presence of premature stop codon and truncated *inlA*. The visualization of the genes profiles and genes presence/absence according to their cgMLST was visualized using the Interactive Tree of Life (iTOL) (https://itol.embl.de/).

The phenotypical AST results are reported in Figure 1 and Supplementary Table S4. All the 18 *L. monocytogenes* isolates showed a resistant phenotypic profile to cephalosporins (cefaclor, cefepime, cefixime, ceftriaxone, and cefuroxime) confirming their natural resistance to this molecule. In the case of penicillin, except one (2021.TE.16), all isolates were susceptible to ampicillin. Two strains were resistant to tetracycline (2021.TE.14 and 2021.TE.16), and finally one strain (2021.TE.11) was resistant to trimethoprim/sulfamethoxazole (miscellaneous agents).

For chloramphenicol, one isolate was susceptible; all the other strains showed a MIC value greater than the maximum value of concentration of this compound in the plate and makes results not interpretable. In the case of quinolones, for sparfloxacin, eight strains were susceptible, while all the other strains showed a MIC value greater than the maximum value of concentration of this compound in the plate making results not interpretable; whereas for levofloxacin, all the strains were susceptible. Finally, all strains were susceptible to ampicillin/sulbactam and amoxicillin/clavulanic acid, carbapenems (imipenem and meropenem) and macrolides (clarithromycin and erythromycin).

Additionally, the identification of antimicrobial resistance genes stated that in all the *L. monocytogenes* isolates tested intrinsic core genes were observed, in particular: *fosX*, *mprF*, *norB*, *sul*, conferring resistance to fosfomycin, quinolones and cationic peptides and sulfonamides, respectively.

### 3.3. WGS and bioinformatic analysis

The results of MLST, cgMLST, virulence, stress resistances and mobile genetic elements profiles were reported in Figure 1. The MLST analysis grouped the 18 *L. monocytogenes* isolates in 7 different Clonal Complexes (CCs): CC1 (*n* = 5), CC2 (*n* = 4), CC9 (*n* = 4), CC5 (*n* = 2), CC121 (*n* = 1), CC155 (*n* = 1), and CC3 (*n* = 1). Data in details are reported in Table 1.

According to the cgMLST results, several clusters were detected. For CC1, 4 of the 5 isolates (2021.TE.2; 2021.TE.3; 2021.TE.4; 2021.TE.11), from ham and salami of M1, showed only 1 AD from each other. One CC1 strain (2021.TE.14) from biltong was a singleton, showing 39 AD from CC1 cluster. Regarding the CC2, three strains (2021.TE.5, 2021.TE.6 and 2021.TE.9) isolated in different products from M2, showed ≤7 AD each other. One polony strain (2021.TE.10) from M5, showed 67 AD from CC2 cluster. For the CC9 strains, 7 AD was highlighted between two strains (2021.TE.16, 2021.TE.19) isolated from polonies of M7. The other CC9 strains (2021.TE.8 and 2021.TE.15), both isolated from sausages showed >7 AD from CC9 cluster. Two *L. monocytogenes* strains (2021.TE.12 and 2021.TE.13) were CC5 showing 3 AD from polonies of M1 and M6. Three strains (one CC121, one CC155 and one CC3) were singleton from 3 different M.

Regarding the virulence factors, all the CCs detected had a complete *L. monocytogenes* Pathogenicity Island 1 (LIPI-1), while a complete LIPI-3 was present both in the CC1 and CC3 isolates. All the CCs carried *vip*, *virR, and virS* genes and a full-length *inl*A for Internalin A and *inl*B for Internalin B. However, only in CC9 and CC121 isolates, a premature stop codon (PMSC) mutation was detected in the *inl*A.

The resistance to stresses, the Stress Survival Islet 1 (SSI-1) was carried by all the CC9, CC5, CC3 and CC155 isolates. Only the CC121 clone, indeed, highlighted the presence of SSI-2. The *bcrABC* cassette was present in 4 CC1 isolates, and in all the CC9 and CC5 *L. monocytogenes* isolates. Only the CC5 and CC155 revealed the presence of *cadA* gene. The *Tn6188*_qac transposon for Benzalkonium Chloride (BC) tolerance was observed in CC121, in one CC1 and CC2 *L. monocytogenes,* together with the *Tn6188*_tetR. The CC121 isolate carried out other transposons (*Tn6188*_tnpA, *Tn6188*_tnpB, *Tn6188*_tnpC).

Regarding mobile genetic elements, plasmids were identified in 9 isolates; the most frequent was plasmid Plm33 (*n* = 5), detected in all the CC9 and in CC3 isolates, followed by plasmid N1-011A (*n* = 2) in CC5 isolates. Plasmids pLM5578 and pLGUG1 were observed only in CC121 and in one CC2 isolates, respectively. Eight different intact prophages regions were found across 17 of the 18 *L. monocytogenes* isolates of this study. The prevalent one was the NC_028871, followed by NC_04990, NC_021539, NC_003216, NC_009813, NC_024387, NC_009815 and NC_028929, reported here as NCBI Reference Sequence.

**Figure 2:**
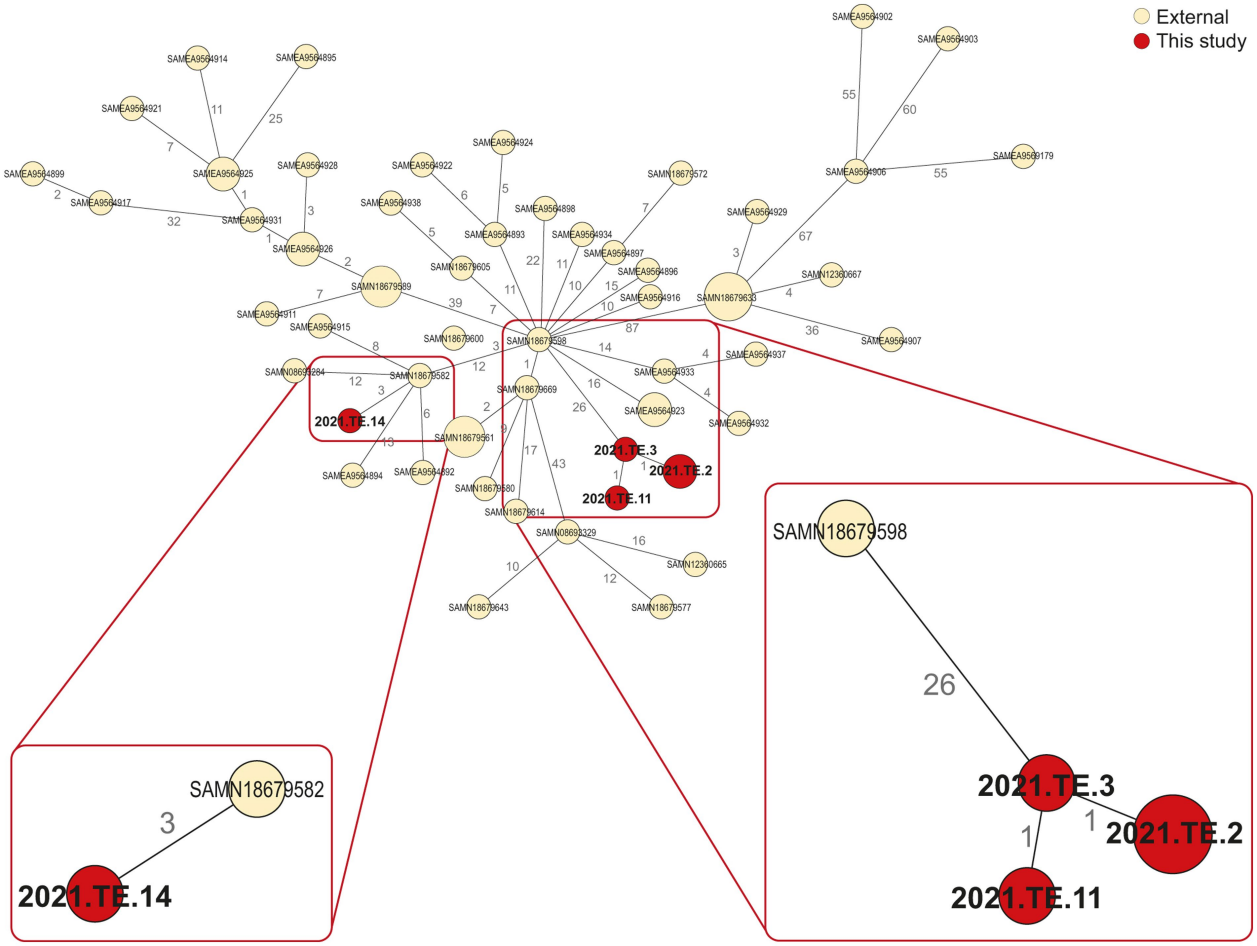
Minimum spanning tree (MST) based on the cgMLST profiles of *L. monocytogenes* CC1. In red were reported the strains from this study, in light yellow were reported the public available genomic data (external) African strains from NCBI. The MST was visualized using GrapeTree (https://github.com/achtman-lab/GrapeTree) and graphically elaborated by Adobe Illustrator to highlight the genomic relatedness (red square).

The comparison between *L. monocytogenes* detected in Zambia and those selected from NCBI, available from SA, showed the presence of several relatedness’s. In regards to CC1 isolates (Figure 2), 3 AD was observed between one CC1 isolate from biltong (2021.TE.14) and one isolated in 2016 from a retail (processed meat-beef) in Gauteng (SA) (SAMN18679582). Similarly, 26 AD was observed between our strain (2021.TE.3) from ham sample and one *L. monocytogenes* isolated from a butchery (processed meat-beef) in Limpopo (SA) in 2016 (SAMN18679598).

**Figure 3:**
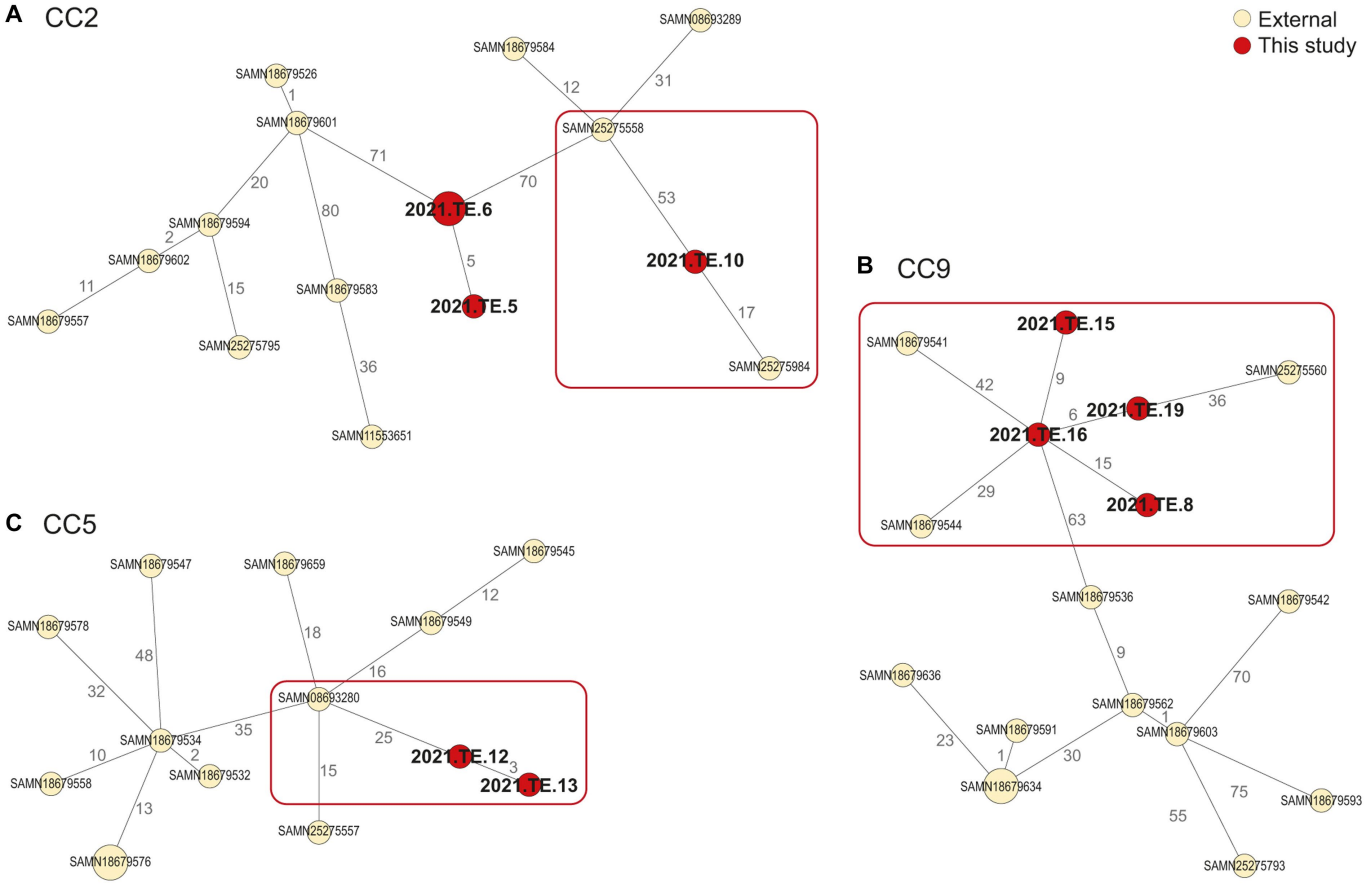
Minimum spanning tree (MST) based on the cgMLST profiles of *L. monocytogenes* CC2 **(A)**, CC9 **(B)**, and CC5 **(C)**. In red were reported the strains from this study, in light yellow were reported the public available genomic data (external) African strains from NCBI. The MST was visualized using GrapeTree (https://github.com/achtman-lab/GrapeTree) and graphically elaborated by Adobe Illustrator to highlight the genomic relatedness (red square).

Regarding the CC2 (Figure 3A), 17 AD was observed between our isolate (2021.TE.10) and one isolated from a retailer in SA, in 2018 (SAMN25275984). For the CC9 (Figure 3B), one isolate (2021.TE.16) from polony showed 29 AD from an isolate detected in 2014, in North West (SA) from retail (raw poultry) (SAMN18679544). Moreover, the same isolate from this study, showed 42 AD from SAMN18679541, isolated in 2015 from Coldstore (Raw-Beef) in Eastern Cape (SA). Another CC9 isolate from this study (2021.TE.19) isolated from polony, showed 36 AD from an isolate SAMN25275560 detected in 2018 in a retailer located in SA. Lastly, for CC5 (Figure 3C), <30 AD was observed between our isolates (2021.TE.12 and 2021. TE.13), both from polony samples, and a clinical isolate from SA (SAMN08693280).

## 4. Discussion

To date, very few data are available on *L. monocytogenes* in RTE foods from Zambia and in Zambian products. Our findings highlighted the presence of *L. monocytogenes* mainly in ham and polony; the latter was the same type of product involved in the SA outbreak (Smith et al., 2019). Previous studies conducted in Zambia reported the prevalence of *L. monocytogenes* in 74% of poultry dressed carcasses (Mpundu et al., 2022a) and 26.3% in cattle carcasses (Mpundu et al., 2022b).

Data are available from African countries, reporting the presence of *L. monocytogenes* in different matrices, such as foods, animal and environmental samples, in Nigeria, SA, Egypt, Ethiopia, and Botswana (Dufailu et al., 2021). In particular data related to *L. monocytogenes* in East Africa, Ethiopia, reported a prevalence of 6.25% in RTE food of animal origin (Garedew et al., 2015), lower than our findings. In Nigeria, a prevalence of 7% was detected in raw meat samples from Rivers State (Odu and Okonko, 2017), while 91.8% in chicken flocks and meat in Oyo (Ishola et al., 2016). In Lafia (Nigeria), the 64.4% of isolates from beef and chevon were confirmed to be *L. monocytogenes* (Chuku et al., 2020). Additionally, in locally made soft cheeses the 12.4% of isolates were *L. monocytogenes* (Oyinloye, 2016). In a study, Matle et al. (2019), conducted in meat samples from SA over several years, reported *L. monocytogenes* in 13.5% of RTE foods collected, higher than the one obtained in our study. The presence of *L. monocytogenes* in the RTE meat placed on markets could be mainly due to inadequate hygiene management systems (Matle et al., 2019), which is a problem in many African countries.

It emerged in this study that several FPP considered presented more than one RTE meat category contaminated by *L. monocytogenes*. The subsequent placing on the market of these contaminated products and their availability for the consumers, poses a high risk especially for vulnerable people. The risk associated with RTE products depends mainly on the effectiveness of control measures applied by food business operators (FBOs), including good hygiene and good manufacturing practices, both at processing and retail (European Food Safety Authority, 2022). In smaller retail store and local manufacturers, inadequate equipment for the preparation of RTE foods may also increase the contamination risk of the final products. In addition, inadequate procedures applied to clean and sanitize FPE (i.e., cutting boards) may increase the cross-contamination of other food products and the persistence of *L. monocytogenes*. Moreover, the presence of *L. monocytogenes* is reported in animals (Heredia and García, 2018), then the contamination of processed foods could be also related to inadequate management of animals, agriculture and farming practices, not only to the post-processing phases.

In literature is well known that listeriosis cases are predominantly associated with RTE foods, including RTE meat products (European Food Safety Authority, 2022). As reported, in the USA, the main listeriosis cases were caused by meat products sliced at retail (Forauer et al., 2021). Food products sliced in retail shops presented a higher bacterial contamination than products prepared in FPP (Forauer et al., 2021), hence RTE food preparation at retail shops could increase the risk of cross-contamination of products from different FBOs (Kurpas et al., 2018).

The WGS approach provided crucial information regarding the CCs distribution and the genomic characteristics of *L. monocytogenes* isolated from Zambian RTE meat foods, especially related to CC1 and CC2 clones, the main CCs detected in the RTE products tested. CC1 and CC2 are both well known to be hyper-virulent clones, often associated with clinical infections (Maury et al., 2016), their presence in RTE foods is a crucial concern for consumer safety. In literature, CC1 clone was often isolated in the pork-meat production sector and it is frequently associated with dairy sector (Félix et al., 2018). The presence of hyper-virulent clones in foods has been reported worldwide, including African countries, suggesting the *L. monocytogenes* adaptation to food products (Matle et al., 2020; Mafuna et al., 2021). The clustering conducted in this study with other public available African *L. monocytogenes* genomes, showed that one CC1 isolate from biltong sample, seems to be closely related to CC1 strain previously detected in 2016 from a SA processed meat.

According to our results, both CC1 and CC2, carried a full-length internalins, *inlA* and *inlB*, which are considered one of the most influent factors for *L. monocytogenes* invasiveness (Su et al., 2019). Increased hyper-virulence and bacterial colonization has been reported in isolates with a full-length *inlA* and a complete LIPI-1 and LIPI-3 (Maury et al., 2016; Yin et al., 2019), suggesting that some isolates have increased virulence potential (Gray et al., 2021). In addition to a complete LIPI-1, CC1 isolates also harbored a complete LIPI-3, encoding the production of Listeriolysin S, a hemolytic and cytotoxic factor also observed to be released in acid stress (Tavares et al., 2020).

Previous studies reported that CC9 and CC121 were often associated with meat products, including RTE, and FPP worldwide, also in African countries (Matle et al., 2020; Mafuna et al., 2021). As known, *L. monocytogenes* is able to resist under stress conditions, supporting its spread in many types of foods (Kurpas et al., 2018). In particular, CC9 and CC121 are well known to be better adapted to meat processing environments (Maury et al., 2019; Centorotola et al., 2021; Guidi et al., 2021b). Specific factors are reported to be crucial for *L. monocytogenes* resistance to the environmental stress. In particular, the presence of SSI-1 ensures resistances to low pH, high osmolarity, bile and nisin, while SSI-2 confers resistances to alkaline and oxidative stresses (Maury et al., 2019). According to our results, SSI-1 was observed in *L. monocytogenes* strains belonging to different clones, whereas the SSI-2 genes were mainly found in CC121 isolates (Harter et al., 2017). The presence of these genes increases the survival, resistance and persistence capacity of *L. monocytogenes* in the FPP, increasing the risk for consumers. In addition, the presence of *Tn6188*_qac transposon confers tolerance to BC disinfectant, mainly used for sanitation in food processing plants. In this study, in fact, CC9 was one of the main clones observed in the RTE meat products tested, and this finding indicates a need for improvement in the FPP sanitation. However, a PMSC mutation was detected in the *inl*A gene, confirming CC9 and CC121 clones as hypo-virulent (Maury et al., 2019; Guidi et al., 2021b). Moreover, *L. monocytogenes* strains showing PMSC in *inl*A were reported in literature, isolated both from clinical case (Magagna et al., 2023) and animal (Sévellec et al., 2020). Our results showed the CC5 as one of the main clones in RTE meat products, as reported also in China (Wang et al., 2015). In the study of Maury et al. (2016), CC5 was not classified as a hyper-virulent clone, but further studies are needed. This clone caused several outbreak cases, such as the cantaloupe case in the USA in 2011 (Lomonaco et al., 2013) and in a previous study by Zhang et al. (2019), the CC5 was reported as the second common clone in clinical *L. monocytogenes* strains in Beijing (China).

Regarding the minor CCs detected in this study, CC155 strains were mostly found in farm and environmental samples, but also this clone was detected worldwide both in food samples and in clinical cases (Matle et al., 2020). *L. monocytogenes* CC3 was reported as prevalent in a study conducted on cooked food tested in China and also in clinical samples (Wang et al., 2018). Similarly to previous data (Chen et al., 2019), and like CC1, the CC3 isolate reported in this study harbored a complete LIPI-3 that could be responsible for the increasing of virulence (Vilchis-Rangel et al., 2019). CC3 strains were also reported in poultry RTE foods during a previous outbreak in SA (Matle et al., 2020).

The comparison between *L. monocytogenes* detected in Zambian RTE and those selected from NCBI, available from SA, showed the presence of several relatedness’s, in particular for hyper-virulent CCs such as CC1 and CC2. This findings was crucial, highlighting how is important to continue to detect and characterize African *L. monocytogenes* strains in order to improve knowledge on strains circulation, surveillance and the monitoring of any possible clusters or new outbreak. About antimicrobial resistance *L. monocytogenes* strains of this study showed to be widely susceptible to clinically relevant classes of antibiotics (penicillin, carbapenems and quinolones) similar to previous reports (Wiśniewski et al., 2022). Our results also confirmed the intrinsic resistance to broad-spectrum cephalosporin antibiotics, commonly used in therapy of bacterial infections (Krawczyk-Balska and Markiewicz, 2016). WGS identification of antimicrobial resistance genes revealed that in all the *L. monocytogenes* tested, intrinsic core genes were observed, as expected, according to what is found in literature (Kurpas et al., 2020). Two strains showed to be phenotypically resistant to tetracycline, despite the absence of *tet* genes. This could be driven by adaptive mechanism such as the antimicrobial resistance determinants exchange with other bacteria through horizontal gene transfer or plasmid mobilization (Wiśniewski et al., 2022). Just one strains showed a resistant phenotype against trimethoprim-sulfamethoxazole concordant with the presence of *sul* gene. However, all the strains resulted susceptible to the antimicrobials used as first choice for the therapy of human listeriosis (Baquero et al., 2020). *Listeria* spp. isolated from different parts of Africa are generally susceptible to ciprofloxacin, but resistant to penicillin (Dufailu et al., 2021). Previous studies reported the increase in resistance of *L. monocytogenes* strains to antibiotics of different classes (Godreuil et al., 2003; Swetha et al., 2021). Keet and Rip (2021) described the presence of isolates susceptible to all antibiotics tested in RTE food, at the same time some isolates were resistant to erythromycin and tetracycline and some strains from polony were multi-drug resistant.

According to literature, the presence of genetic mobile elements, such as plasmids and prophages, increases the *L. monocytogenes* resistance. Interestingly, several studies indicated that some plasmids might confer increasing tolerance to stresses, heavy metals, quaternary ammonium compounds, salt, oxidative, heat, and cold stress (Mafuna et al., 2021; Schmitz-Esser et al., 2021). According to previous studies, among the hyper-virulent clones, few strains carried plasmids; on the contrary, the hypo-virulent clones normally show a higher number of plasmids (Schmitz-Esser et al., 2021). With the additional presence of disinfectant resistances, such as the *bcrABC* cassette and the *Tn6188*_qac for BC tolerance, various prophages and plasmids involved in stress response, the hyper-virulent clones could resist several stresses and probably persist in the environment for years. Data from literature highlighted the distribution of the hyper-virulent clones and their ability to contaminate, during the years and for long time, food products, food processing plants in Africa, increasing risk for human (Mafuna et al., 2021).

## 5. Conclusion

Although there has been listeriosis cases reported worldwide, *L. monocytogenes* data from Africa remains limited. Despite being a first preliminary work with a limitation in the sampling plan and a limited number of samples and isolates, this study reported our findings providing justification for a more complete surveys in future work, and helping to increase existing knowledge on contaminated RTE products circulating in Africa countries. Furthermore, this study highlighted interesting results about *L. monocytogenes* in Zambian RTE meat foods, confirming the presence of hyper-virulent CCs, which could play an important role in human diseases, posing a public health concern for consumers. Moreover, the presence of stress resistance factors could help these hyper-virulent clones to adapt, survive and persist over the years, as described by relatedness found in this study with other *L. monocytogenes* isolated in SA, during the years, from different matrices.

This preliminary study was conducted in order to test local Zambian foods available in local supermarkets, but also to allow generalizable findings. Further studies should be conducted on RTE products from Zambia and other African country, in order to deeply investigate the worldwide diffusion and the genomic characteristics of *L. monocytogenes* strains with particular attention on hyper-virulent clones aiming to improve worldwide surveillance and food safety.

In the future it will become crucial to improve the sampling plan, test more RTE foods and food environmental samples, but also to collect and characterize by WGS more *L. monocytogenes* isolates, especially from each positive sample, improving data and knowledge on *L. monocytogenes* in food products from Africa.

Due to the global increasing request, trade and consumption of foreign foods, it would be necessary to improve hygiene conditions and standardize procedures for hygiene and sanitation in food processing plants in Africa countries. Moreover, it could be useful to apply a better practices related to animal, agriculture and farming management in order to reduce *L. monocytogenes* in preharvest animals and consequentially in RTE meat products. According to One Health approach, it will be important compare available *L. monocytogenes* WGS data with clinical strains, in order to identify any clusters, including any potential connections to listeriosis cases and outbreaks.

## Data availability statement

The datasets presented in this study can be found in online repositories. The names of the repository/repositories and accession number(s) can be found in the article/Supplementary material.

## Author contributions

MZ, GC, GM, and FP conceptualized the study. MZ, BB, SM, and PM carried out sample collection, identification and isolation experiments. GC, ACo, ACh, and CC carried out the WGS experiment. ACh and GC carried out the bioinformatics analysis. GC, MZ, ACo, and ACh analyzed the data, organized the draft, and wrote the manuscript. FP, MT, and GM supervised the entire work. ND’A and MS acquired the funding. GC, MZ, ACo, ACh, MT, FG, CC, BB, SM, PM, ND’A, MS, FP, and GM contributed to editing the manuscript and reviewed the final version. All authors have read and approved the manuscript.

## Funding

This work was supported financially by “Enhancing Research For Africa Network” (ERFAN) project, Italy, in collaboration with the Ministry of Fisheries and Livestock, Zambia.

## Conflict of interest

The authors declare that the research was conducted in the absence of any commercial or financial relationships that could be construed as a potential conflict of interest.

## Publisher’s note

All claims expressed in this article are solely those of the authors and do not necessarily represent those of their affiliated organizations, or those of the publisher, the editors and the reviewers. Any product that may be evaluated in this article, or claim that may be made by its manufacturer, is not guaranteed or endorsed by the publisher.

## Footnotes

^1^https://assets.thermofisher.com/TFS-Assets/MBD/brochures/Sensititre-Plate-Guide-Booklet-EN.pdf

^2^https://bigsdb.pasteur.fr/

^3^https://phaster.ca/

^4^https://cge.food.dtu.dk/services/PlasmidFinder/
