# Supplementary material for: Listeria monocytogenes in ready to eat meat products from Zambia: phenotypical and genomic characterization of isolates
Source: Front Microbiol. 2023 Aug 30;14:1228726. doi: 10.3389/fmicb.2023.1228726 (PMC10498467; doi:10.3389/fmicb.2023.1228726)
Supplement: Supplementary file 1 [file Data_Sheet_1.zip › Supplementary tables captions.DOC]

Supplementary Material

# Supplementary material captions:

**Table S1:** Sample identification, product type, ready to eat category, data of sampling and food business operators related to the 304 samples considered in the study.

**Table S2:** Minimum inhibitory concentration (MIC) breakpoint for each antimicrobial tested for the 18 *L. monocytogenes* strains.

**Table S3:** Dilution range of antimicrobials tested for the 18 *L. monocytogenes* strains in HPB1 plate.

**Table S4:** Phenotypic antimicrobial susceptibility testing results of 18 *L. monocytogenes* strains tested.
